# Book Review

**Published:** 1928

**Authors:** 


### Reviews of Books.

**The Thomas Splint.** By Meurice Sinclair, C.M.G., M.B. Pp. xiv., 168. 85 illustrations and 3 charts. Oxford University Press. 1927. Price 15s.—The present short treatise gives a very good account of these methods and enables the reader to understand both the principles of treatment and the details of construction of the apparatus. Probably it is an exaggeration to say that the lessened mortality of the gun-shot fractures in the war was chiefly owing to the adoption of the Thomas Splint. It was the segregation of the cases under the care of special officers and the establishment of team work which was the great secret of improvement of results. But now that the value of Thomas's methods is generally recognised, it is of great value to have a small and clearly written book like the present giving all the technical directions necessary for their efficient application.

**Fatalism or Freedom: a Biologist's Answer.** By C. Judson Herrick. 12mo. Pp. 106. London: Kegan Paul, Trench, Trübner. 1927. Price 2s. 6d.—Are we free to control our conduct or is our behaviour rigidly predestined and beyond our power to alter? This perennial problem is once more attacked, this time from the standpoint of a biologist. The author first considers biological control, and says: "Every organism to some extent controls the environment in which he lives, and the pattern of this control of environment through behaviour, is shaped by the internal organisation of the individual which, in its turn, has grown up during the exercise of these distinctly vital functions." This control is at its highest in man because of his greater variety of response in behaviour, due to the more complex structure and varied function of his nervous system. Similarly the control exercised by mental processes, thoughts, feelings, volitions, etc., is an expression of the function of the highly complex and specialised brain and glandular organs of man. No scientific exposition can allow of a mystical freedom unrelated to cause and effect, but we can talk of a natural freedom related to definite causal agencies. This natural freedom means that a thing or process with specific organisation is able to exhibit the behaviour characteristic of or typical for that organisation in an appropriate environment. This freedom must appear within the lawful system not opposed to it, its value depends on the pattern of the organisation, and the setting or total situation within which it operates. The powers of reflective thought and prevision are important factors in influencing the organisation within which the human being is free, yet his choice is none the less causally determined by the organisation of his reflexes, instincts and sentiments. We may, therefore, say that " the normal man in the normal environment is free to work out his own inner nature, and to enlarge and refine it." He has power to direct his bodily energies through different channels, and we may see that these choices are in accordance with natural law, though we may not be able at our present stage of knowledge to exactly state these laws. This concept of freedom must appeal to those who regard science as the method of approach to all the problems of life, and this little book may be confidently recommended to clarify a difficult subject. It amply maintains the high level of Psyche Miniatures on which the publishers are much to be congratulated.

**The Nature of Disease, Part II.** By J. E. R. McDonagh, F.R.C.S. Pp. vi., 434. 128 figures. London : Wm. Heinemann Ltd. 1927. Price 21s.—Those who have read and appreciated Part I. of this series will welcome Part II. They will remember that in the former the thesis was announced that all disease is one ; as it were, "There is no disease but Disease (and McDonagh is the exponent of Disease)." The changing aspect of disease from case to case was explained in terms of the essential humours ; it is true that in place of invoking phlegm or melancholy, one was told that the protein particles of the plasma were in a molecular dispersoid state, or that the viscosity of the serum was abnormal. The main theme of the present volume is chronic intestinal intoxication, the author's views on focal sepsis being frankly those of the " autotoxis " school of the nineties. If a patient has infected tonsils, or a dental granuloma, it is intestinal sepsis that has produced this. Three hundred pages are devoted to the discussion of this subject, with some one hundred and fifty elaborate case histories. In nearly all, treatment included colon lavage and the administration of substituted symmetrical urea compounds ; though since most had much other treatment, as operations, vaccines, dieting, contramine, thyroid, insulin, etc., the issue is somewhat obscured. It is distressing to read, " The patient was subjected to every kind of treatment, and in spite of the blood picture having been brought back to a normal condition, no clinical improvement could be registered." The remainder of the book is devoted to an explanation of the action of drugs, vaccines and so forth, on a "rational" as opposed to an "empirical" basis, illustrated by means of the author's suggested electronic formulæ for over a hundred preparations. These occupy much space, some of them taking a whole page each; but the average medical reader will be moved rather to awe than to understanding. An appendix on animal experiments and a voluminous index concludes the book; but we are encouraged by the prospectus of Part III. to await its appearance with what patience we can muster.

**Tonic Hardening of the Colon.** By T. STACEY WILSON, M.D., F.R.C.P. Pp. xxiv., 210. London: Oxford University Press. 1927. Price 8s. 6d.—That this terrible disorder has not attracted the attention it merits is ascribed by the author to a failure to recognise its possibilities. The symptomatology is wide and varied. Cardiac, cardio-vascular and circulatory changes; respiratory irregularities and embarrassment; digestive disorders of the most diverse types, but especially those suggestive of gastric or duodenal ulcer; and, finally, every form of mental depression from slight feelings of "lowness" to suicidal impulses; may all be indicative of tonic hardening of the colon. "The term Tonic Elastic Fixation may be used for this non-plastic form of static activity as it manifests itself in the colon." On palpation, the colon is felt to harden up under the hand, and to be tender; and a very large proportion of the author's patients show this sign. Fortunately, diagnosis is comparatively easy, and treatment simple, consisting of intestinal antisepsis, antispasmodic drugs, and avoidance of constipation. So that possibly many physicians have been treating the condition for years on the symptomatology, without ever recognising the real cause of their patients' troubles. We are warned, however, that it does not follow that because the patient has this complaint he has no other; so that if failure attends our efforts to relieve we must be prepared to look farther afield, to operative measures on the one hand, or to psycho-analysis on the other. The author gives full directions for the diagnosis and treatment of the disease, with an account of the physiology and pathology of the sympathetic control of the bowel, and numerous case histories, to which reference is facilitated by an adequate index.

**The Tongue and its Diseases.** By DUNCAN C. L. FITZWILLIAMS, C.M.G., M.D., Ch.M., F.R.C.S. Pp. xvi., 505. Illustrated. Oxford University Press. 1927. Price 36s.—We review this book with much pleasure, for it is very creditable to the author, and is evidence of an immense amount of research into the literature and experience of the subject. He describes the normal, comparative and pathological anatomy of the tongue, and the appropriate treatment of its diseases, with abundant extracts of case-reports from numerous sources. It is destined to become a standard work of reference. The importance of cancer and its treatment necessitates a summary of his views. We agree with his objection to preliminary ligation of the lingual arteries, and he argues reasonably against removing the glands before the tongue, intratracheal anæsthesia, and the preliminary administration of morphia except in very small doses. Of diathermy, he thinks it has few advantages, and serious disadvantages over the ordinary operation, although useful in less accessible situations. Many of the illustrations are excellent, but we think Figures 27, 46, 61, 65 and 106 could be improved in a future edition, for they fail to represent what is intended ; and the description of cancrum oris (page 135) might be revised. We commend the book very heartily, and congratulate the author on the way he has studied the copious literature.

**Pneumothorax and Surgical Treatment of Pulmonary Tuberculosis.** By CLIVE RIVIERE, M.D., F.R.C.P. Second Edition. Pp. xxii., 311. 9 illustrations, 16 plates. Oxford University Press. 1927. Price 10s. 6d.—"No more hopeful ray of sunshine has ever come to illumine the dark kingdoms of disease than that introduced into the path of the consumptive through the discovery of artificial pneumothorax." Yet it is extraordinary to think of the profound ignorance of many tuberculosis officers with regard to this proven form of treatment. This book clearly sets out the indications and contra-indications. A full account is given of cases treated, and the surgical operations at present in use are discussed. Dr. Riviere is an enthusiast, he is strongly against too early interference, but thinks it a pity that the intermediate period is so often let pass, and that the method is far too often used as a last resort. In such cases the mortality is necessarily high. We feel that the author fails to lay sufficient stress on the importance of sanatorium regime as an adjuvant ; and might give a more detailed explanation of the skiagrams. Though there are other debatable points, this book ought to be in the library of everyone, physician or surgeon, interested in the treatment of pulmonary tuberculosis.

**Idiosyncrasies.** By Sir HUMPHRY ROLLESTON, Bart., K.C.B., F.R.C.P. Pp. 119. London: Kegan Paul, Trench, Trübner. 1927. Price 2s. 6d. This is a small monograph which comprises within a space of about one hundred pages a brief tabulation of the various forms of "idiosyncrasies." The meaning of the word is discussed, and its association with psychic and physical states, the action of foods and drugs, and their reaction upon the skin and mucous membranes, etc. The book is eminently readable and comprehensive, as all of Sir Humphry Rolleston's writings are, and suggests some new ideas with regard to the possible association of gout and rheumatoid arthritis with allergy.

**Aspects of Rheumatism and Gout.** By L. J. LLEWELLYN, M.B. Pp. xiii., 295. London: William Heinemann. 1927. Price 10s.—With his usual masterly style, the author discusses these maladies from his own view-point, bringing out many interesting features, the result being a veritable classic of clinical philosophy. He is at great pains to expound the doctrine of diathesis, and in the chapter concerned with this matter brings forward many interesting and acceptable facts, showing a wide knowledge of biological detail. As suggested in the foreword, there is considerable overlap in several of the chapters, the constant reiteration of the theory of endocrine imbalance tending eventually to hypnotise the reader into acceptance. Even so, a good case is made out from this standpoint as regards the pathogeny of the rheumatic group of diseases—there is in the author's mind serious doubt as to the infective element in its ætiology, "its pathogeny cannot be pent up in the narrow compass of a septic tooth"—for him it results from a combination of factors, amongst which thyroid deficiency is to the fore, hand in hand with local metabolic disturbances. His discussions on the pitfalls of diagnosis are illuminating. The chapter on the prevention of rheumatism is full of sound judgment and of recommendations which should be of great benefit were they efficiently carried out. As regards gout, diathesis or individual susceptibility to external influences is again the keynote—"in gout, as in asthma, every man is a law unto himself." Altogether, a work promoting deep reflection, and inviting one to view the rheumatic group from a standpoint rather different from that which is customary.

**Diseases of the Lungs.** By F. E. TYLECOTE, M.D., F.R.C.P., and G. FLETCHER, M.D., M.R.C.P. Pp. viii., 270. London: Oxford University Press. 1927. Price 7s. 6d.—This is a comprehensive, well written little work, the authors showing throughout great practical knowledge of the subject. Their remarks on breath sounds and adventitious sounds are particularly happy and informative, and they indicate how the various types of breath sounds are really modifications of the sounds produced at the glottis, varying according to the conductivity of the lung tissues. These facts are usually not stressed enough in the ordinary text-books, the student thereby failing to understand the true significance of alterations in the respiratory murmur. A belated reference only is made to the treatment of bronchitis by vaccines, and not much faith is placed in this line of attack on bronchiectasis—under the ætiology of the latter condition, while it is claimed that one of the factors is an increased air pressure within the bronchi produced by coughing, it does not seem to be recognised that, given an intact thorax, there is a corresponding increase in pressure outside and around the bronchi. The rôle of anaphylaxis in the ætiology of asthma does not seem to be elevated to the prominence which it deserves, and treatment with peptone injections does not appear to find great favour; the antispasmodics and potassium iodide are the sheet anchors of general treatment. The diagnoses of empyemata and thoracic tumours are well discussed. The last chapter is devoted to pulmonary tuberculosis, the diagnosis of which is handled on sound common-sense principles, and the treatment and management of a case in a likewise commendable way. This book, presenting as it does certain facts in a way to make them the more easily appreciated, should certainly, as suggested in the preface, be most helpful and acceptable to the student and practitioner.

**The Injection Treatment of Varicose Veins.** By A. H. DOUTHWAITE, M.D., M.R.C.P. Pp. viii., 39. London: H. K. Lewis & Co., Ltd. 1927. Price 3s. net.—Though rarely a source of serious alarm or trouble, the cumulative effect of chronic pain from varicose veins cripples the nation's workers considerably. This disability exists despite our present means of treatment. In *The Injection Treatment of Varicose Veins* there seems a promise of great reductions in this silent suffering, borne largely by the weaker sex. Not that obliteration of varicose veins by chemical phlebitis is more effective than by operation, but its advantage is that the treatment does not interfere with the patient's ordinary life, as the author says. His opinion is based on 2,000 cases. Such experience, and the lucid and scientific handling of his subject, make the book a good guide to a new remedy.

**Social Factors in Medical Progress.** By BERNHARD J. STERN, Ph.D. Pp. 136. New York: Columbia University Press. 1927. Price $2.25.—This volume forms one of a series of studies in history, economics and public law, edited by the Faculty of Political Science of Columbia University. The author divides his subject into two parts, of which the first deals with factors which retard the diffusion of innovations, and the second with the nature of progress in medicine. The retarding factors include vested interests, the power of tradition (always intensified by educational systems !), and reverence for authority. Dr. Stern gives interesting illustrations of the way in which even really great scientists may oppose novel scientific discoveries, instancing Virchow's refusal to see anything in Charcot's ataxic joint symptoms, and Simpson's opposition to Lister's discoveries. He has a short chapter on the opposition to dissection, full of historical peeps. He goes on to deal with the opposition to Harvey, to Auenbrugger, to Jenner, to the views of Holmes and Semmelweiss on puerperal sepsis, to Pasteur and to Lister. In the second part Dr. Stern is inclined to reject the idea that progress in medicine is represented by a series of wonderful jumps from one individual genius to another, and raises the question whether the discovery would not have been made irrespective of the individual who is now heralded as the discoverer. This is a delightful book, and none the less so for the solace it brings to the humbler folk who have never discovered anything.

**Dermatological Neuroses.** By W. J. O'DONOVAN, O.B.E., M.D., F.R.C.P. Pp. 99. London: Kegan Paul, Trench, Trübner. 1927. Price 2s. 6d.—In this short treatise an attempt is made to urge the "psychological element" in dermatological cases. Instances are adduced in which a psychic factor was plainly of importance in estimating the ætiology, and in directing the treatment of various dermatoses. So often is this psychic factor of paramount importance in the chronic pruritic dermatoses, that O'Donovan states that "the queue of waiting cases of lichen, chronic urticaria, and of half-diagnosed cases of eczema will be shortened if the emotional tone and psychological history of the patient are taken into account and turned to the patient's advantage."

**Problems in Psychopathology.** By T. W. MITCHELL, M.D. Pp. 190. London: Kegan Paul, Trench, Trübner & Co. 1927. Price 9s.—This book consists of lectures delivered before the British Institute of Philosophical Studies, chiefly upon the Freudian teaching. Dr. Mitchell regards psychology as a science of mind with no necessary relation to the physiology or anatomy of the nervous system. On this basis he states that medical psychology must confine itself to the description, classification and treatment of abnormal mental states. He deals first with hypnosis and hysteria, regarding them as phenomena of dissociation, which is the basis of Janet's theory of the neuroses, but Dr. Mitchell properly criticises this as giving no explanation as to why dissociation occurs in the particular way which we observe in any given patient. The explanation of this pathological process is to be found in the work of Freud, and most of the book is taken up in a review of Freud's doctrine, with more particular reference to its most recent development. He discusses the development of the ego and the super ego from the id, the differentiation of the life and death instincts and the ultimate analysis of the ego, as Freud deals with these problems. However, even Dr. Mitchell's customary lucidity does not make these theories very readily comprehensible; and since Freud takes no cognisance of other theories of instincts, or indeed of other teachers of psychology, comparison with his contemporaries is of no service whatever. It is claimed that these theories elucidate the behaviour of psychotic patients; but, because Freud has, or thinks he has, formulated a complete explanation of the psychoneuroses, we may ask whether he is justified in applying the same explanation to the psychoses as if the problem was entirely the same. Finally, Dr. Mitchell refers to divergencies from the psycho-analytic theory, and seems to regard them with scant respect. For those who wish to find a clear exposition of psycho-analytic doctrines presented in a relatively small compass, and in language as clear and simple as is compatible with scientific exactitude, the present volume may be confidently recommended.

**Diathermy.** By E. P. CUMBERBATCH, M.R.C.P. 2nd edtion. Pp. xiv., 332. 87 illustrations. London: William Heinemann Ltd. 1927. Price 21s.—The issue of a new and revised edition of this work is welcome in view of the rapid progress made in recent years in the medical and surgical uses of diathermy. It is probably the best book on the subject in the English language, and thoroughly up to date, both in the technical and medical sections. The experience gained at St. Bartholomew's Hospital in the treatment of diseases peculiar to women and in gonococcal infections is fully dealt with, the part dealing with the surgical uses of diathermy has been enlarged, and an introductory account of the new "cutting currents" has been added. The print is good, and the book is well illustrated and indexed.

**Exposures of Long Bones and other Surgical Methods.** By ARNOLD K. HENRY, M.B., B.Ch., F.R.C.S.I., Professor of Clinical Surgery, Cairo. Pp. xii., 80. 51 illustrations. Bristol: John Wright & Sons, Ltd. 1927. Price 10s. 6d.—The first part of this small work deals with the surgical and anatomical problems of the complete exposure of the long bones. It is shown how this can be done so as to get the whole shaft of each bone laid bare with the least possible damage to the overlying soft structures. The methods employed in the case of the humerus, radius and femur are of great practical value, especially in those operations, *e.g.* bone grafting, where a wide exposure is essential to success. The other chapters deal chiefly with difficult anatomical problems in relation to surgical operations, *e.g.* the exposure of the plantar structures, a method of ligating the second stage of the vertebral artery, ligation of the first part of the left subclavian artery, resection of the left cervico-dorsal ganglion of the sympathetic and the approach to the pituitary gland under radiographic control. The work is a very good illustration of the value of a close association between anatomy and surgery. The new type of aneurism needle and the "pituitary gun" both display great mechanical ingenuity.

**Outlines of Dental Science. V.: Dental Radiography.** By C. A. CLARK, L.D.S. Pp. 97. Illustrated. Edinburgh: E. & S. Livingstone. 1927. Price 7s. 6d.—This little book takes its natural place in a very useful series of publications. Dental radiology has become part of the present-day student's routine study. This volume has the subject-matter concisely put together. It clearly outlines the apparatus used, and explains in a delightfully short manner the technique involved, both with the patients and the dark room. The various illustrations throughout the book very clearly bring home to the reader the points touched on in the various chapters. The writer wisely concludes his remarks with a chapter devoted to "Interpretation from the Negative."

